# Role of Serum Growth Factor and Inflammatory Markers in Predicting the Progression of Oral Squamous Cell Carcinoma: A Clinical Study

**DOI:** 10.7759/cureus.109594

**Published:** 2026-05-25

**Authors:** Tejal Patil, Jasmine Kalsi, Mukesh Kumar, Rahul VC Tiwari, Ambili Remesh, Unni J Appilly

**Affiliations:** 1 Department of Oral and Maxillofacial Surgery, Bharati Vidyapeeth (Deemed to be University) Dental College and Hospital, Navi Mumbai, IND; 2 Department of General Surgery, Kalpana Chawla Government Medical College, Karnal, IND; 3 Department of Oral and Maxillofacial Surgery, Shree Bankey Bihari Dental College, Ghaziabad, IND; 4 Department of Oral and Maxillofacial Surgery, Narsinhbhai Patel Dental College and Hospital, Visnagar, IND; 5 Department of Pharmacology, Sree Uthradom Thiurnal Academy of Medical Sciences, Trivandrum, IND; 6 Department of Oral Rehabilitation, University of Otago, Dunedin, NZL

**Keywords:** epidermal growth factor receptor, interleukin-6, oral squamous cell carcinoma, prognosis, tumor necrosis factor-alpha

## Abstract

Background

Oral squamous cell carcinoma (OSCC) is a common malignancy with variable clinical outcomes and a high risk of recurrence. Conventional prognostic indicators often fail to fully capture tumor behavior. Serum growth factors and inflammatory mediators, such as the epidermal growth factor receptor (EGFR), interleukin-6 (IL-6), and tumor necrosis factor-alpha (TNF-α), may provide additional insights into disease progression and prognosis. This study aimed to evaluate the prognostic significance of serial serum levels of EGFR, IL-6, and TNF-α in patients with OSCC.

Methodology

This prospective cohort study included 120 patients with histopathologically confirmed OSCC. Serum samples were collected at baseline, 6 months, and 12 months and analyzed for EGFR, IL-6, and TNF-α using enzyme-linked immunosorbent assay. Clinical and pathological data were also recorded. Statistical analyses were performed. Intergroup comparisons were conducted using independent t-tests, correlations were assessed using Spearman’s coefficient, and Cox proportional hazards regression analysis was used to identify predictors of recurrence-free survival.

Results

In total, 34 (28.33%) patients with recurrence showed significantly higher baseline levels of EGFR (82.3 ± 24.1 vs. 62.9 ± 19.4 ng/mL), IL-6 (26.4 ± 10.8 vs. 15.9 ± 7.1 pg/mL), and TNF-α (42.7 ± 16.3 vs. 28.6 ± 12.1 pg/mL) compared to non-recurrent cases (p < 0.001). Similar trends persisted at 6 and 12 months (p < 0.001). Significant positive correlations were identified between baseline biomarker levels and clinicopathological parameters. Tumor size showed moderate correlations with EGFR (r = 0.54), IL-6 (r = 0.48), and TNF-α (r = 0.42). Similarly, tumor-node-metastasis stage demonstrated strong correlations with EGFR (r = 0.61), IL-6 (r = 0.57), and TNF-α (r = 0.51). Lymph node metastasis also showed significant positive correlations with EGFR (r = 0.52), IL-6 (r = 0.49), and TNF-α (r = 0.45) (p < 0.001). Multivariate analysis identified the baseline EGFR (p < 0.001), IL-6 (p = 0.002), and TNF-α (p = 0.028) as independent predictors of recurrence of OSCC.

Conclusions

Serum EGFR, IL-6, and TNF-α may serve as potential prognostic indicators for OSCC. The findings of this study suggest that combining growth factors and inflammatory biomarkers could have clinical utility for improving prognostic assessment in OSCC. Serial monitoring may provide a dynamic approach for identifying patients at higher risk of recurrence, thereby facilitating timely therapeutic interventions and closer surveillance. Incorporation of these markers into routine clinical protocols might contribute to more personalized treatment strategies and potentially improve long-term outcomes. However, further large-scale studies are required to validate these findings and establish standardized clinical thresholds.

## Introduction

Oral squamous cell carcinoma (OSCC) is one of the most common malignancies of the head and neck region and continues to represent a major public health concern, particularly in developing countries with a high prevalence of tobacco and alcohol use [1]. Despite advancements in surgical techniques, radiotherapy, and systemic therapies, the overall prognosis of OSCC remains suboptimal, with the reported five-year survival rate remaining around 50-60%, particularly in patients diagnosed at advanced stages [2]. This is largely attributed to late-stage diagnosis, tumor heterogeneity, and unpredictable patterns of disease progression and recurrence [3]. Therefore, identifying reliable prognostic indicators is critical for improving risk stratification and guiding individualized treatment strategies [4].

Tumor progression in OSCC is a complex process influenced not only by genetic alterations but also by interactions within the tumor microenvironment [5]. Among the key molecular contributors are growth factor-related pathways and pro-inflammatory cytokines, which play significant roles in tumor proliferation, angiogenesis, immune evasion, and metastatic potential [6]. Epidermal growth factor receptor (EGFR) has been widely implicated in tumor growth and resistance to therapy, whereas cytokines such as interleukin-6 (IL-6) and tumor necrosis factor-alpha (TNF-α) are associated with systemic inflammation and tumor aggressiveness [7,8]. Elevated circulating levels of these factors have been linked to advanced disease stages and poor clinical outcomes.

Although several studies have evaluated individual biomarkers such as EGFR, IL-6, and TNF-α in OSCC, most investigations have relied on single baseline measurements and cross-sectional analyses. Such approaches may not adequately reflect the dynamic biological changes occurring during disease progression, treatment response, or recurrence. In addition, limited studies have simultaneously assessed both growth factor and inflammatory markers in a longitudinal setting, and the prognostic significance of their serial monitoring over time remains insufficiently explored. Therefore, there is a need for prospective studies evaluating temporal changes in these circulating biomarkers to better understand their potential role in predicting recurrence and clinical outcomes in OSCC [7-10]. Longitudinal evaluation of circulating growth factors and inflammatory markers may provide a more dynamic and clinically relevant understanding of disease behavior, particularly in predicting recurrence and survival outcomes. This study aimed to evaluate the role of serum EGFR, IL-6, and TNF-α as prognostic indicators in patients with OSCC. The objectives of this study were to assess their association with clinicopathological parameters, compare their levels between patients with and without recurrence, and determine their significance in predicting disease-free survival and overall clinical outcomes.

## Materials and methods

Study design and setting

This study was designed as a prospective observational cohort study and was conducted in the Department of Oral and Maxillofacial Surgery, Narsinhbhai Patel Dental College and Hospital, Visnagar, India, from April 2022 to December 2023. The study was conducted in accordance with the Strengthening the Reporting of Observational Studies in Epidemiology (STROBE) guidelines for observational studies to ensure methodological robustness and transparency [11]. Ethical approval was obtained from the institutional ethics committee before the commencement of the study (approval number: NPDCH/IEC/2022/163). Written informed consent was obtained from all participants before enrollment.

Sample size estimation

The sample size was calculated using G*Power (version 3.1; Heinrich Heine University, Düsseldorf, Germany) based on Cox proportional hazards regression modeling. Considering a five-year recurrence rate of 35% as given in the reference study, a hazard ratio of ≥1.8, an alpha error of 0.05, and a power of 80%, a minimum of 102 evaluable participants were required [12]. Accounting for a 15% attrition rate, a total sample size of 120 patients was included, ensuring adequate events per variable for multivariable analysis.

Study population

In total, 120 patients with histopathologically confirmed OSCC were recruited for this study. Adult patients (≥18 years) with newly diagnosed disease across all stages (I-IV) and subsites of the head and neck region were included [13]. Patients with prior malignancies, systemic inflammatory or autoimmune disorders, chronic infections (such as HIV, hepatitis B/C, or tuberculosis), prior head and neck radiotherapy or chemotherapy, pregnancy, or inability to provide informed consent were excluded from the study. This ensured the minimization of confounding factors that could influence systemic biomarker levels.

Serum sample collection and processing

Peripheral venous blood (10 mL) was collected at baseline (before treatment) and at 6, 12, and 24 months follow-up using serum separator tubes (BD Vacutainer®, Becton, Dickinson and Company, Franklin Lakes, NJ, USA). The samples were allowed to clot at room temperature (20-250°C) for 30 minutes and centrifuged at 1,200 × g for 15 minutes at 40°C to preserve cytokine integrity. Serum was aliquoted into sterile polypropylene cryovials (Eppendorf SE, Hamburg, Germany) and stored at ≤−80°C until analysis. Hemolyzed, lipemic, or icteric samples were excluded to maintain analytical accuracy, and repeated freeze-thaw cycles were strictly avoided.

Biomarker quantification

Serum concentrations of EGFR, IL-6, and TNF-α were determined using a standardized sandwich enzyme-linked immunosorbent assay (ELISA). EGFR was quantified using the Quantikine® Human EGFR ELISA kit, while IL-6 and TNF-α were measured using DuoSet® ELISA kits (R&D Systems, Minneapolis, MN, USA), following the manufacturer’s recommended protocols. All samples were analyzed in duplicate to ensure analytical reliability and reproducibility. For each assay plate, a seven-point standard calibration curve was constructed. Absorbance readings were obtained at 450 nm using a microplate reader (BioTek Synergy H1; BioTek Instruments Inc., Winooski, VT, USA). Assay precision was maintained with intra-assay and inter-assay coefficients of variation below 8% and 12%, respectively, which were within the acceptable limits specified by the manufacturer. The methodology employed is consistent with previously reported studies evaluating circulating cytokines and growth factors in OSCC [14,15].

Data collection and follow-up

Clinical and demographic data were collected using a standardized electronic database implemented through the Research Electronic Data Capture (Vanderbilt University, Nashville, Tennessee, USA). Variables included age, sex, tobacco and alcohol consumption, tumor site, TNM staging, histological grade, and treatment modalities. Follow-up assessments were conducted every three months during the first two years and every six months thereafter, with recurrence-free survival analysis based on follow-up data available up to 12 months after baseline biomarker assessment.

Statistical analysis

Statistical analysis was performed using SPSS Statistics version 26.0 (IBM Corp., Armonk, NY, USA). Categorical variables are expressed as frequencies and percentages, while continuous variables are presented as mean ± standard deviation. Data were analyzed for normal distribution by the Shapiro-Wilk test. Intergroup comparisons were performed using independent t-tests. As the primary objective was to evaluate the prognostic significance of biomarker levels at predefined clinically relevant follow-up intervals and their association with recurrence outcomes, biomarker measurements at each time point were analyzed independently rather than through repeated-measures modeling. Correlations between biomarker levels and clinicopathological parameters were assessed using Spearman’s rank correlation coefficient. Survival analysis was performed using Cox proportional hazards regression to identify independent predictors of recurrence. Hazard ratios (HRs) with 95% confidence intervals (CIs) were calculated. Statistical significance was set at a two-tailed p-value <0.05. All analyses were conducted following a predefined statistical plan to minimize bias.

## Results

A total of 120 patients with OSCC were included, with a mean age of 54.7 ± 11.3 years and a male predominance. Most patients were older than 40 years and had a history of tobacco and alcohol use. Tongue and buccal mucosa were the most common tumor sites. Advanced-stage disease (stages III-IV) was observed in 70 (58.4%) cases, and 76 (63.3%) cases demonstrated moderate-to-poor differentiation. Combined treatment modalities were most frequently employed (Table 1).

**Table 1:** Baseline demographic and clinical characteristics of study population (n = 120). Values are presented as number (percentage). Percentages may not total 100 due to rounding. TNM: tumor-node-metastasis staging system

Parameters	Category	n (%)
Age (years)	≤40	32 (26.7)
	>40	88 (73.3)
Sex	Male	89 (74.2)
	Female	31 (25.8)
Tobacco use	Yes	78 (65.0)
	No	42 (35.0)
Alcohol use	Yes	61 (50.8)
	No	59 (49.2)
Tumor site	Tongue	52 (43.3)
	Buccal mucosa	38 (31.7)
	Floor of mouth	18 (15.0)
	Other	12 (10.0)
TNM stage	Stage I	18 (15.0)
	Stage II	32 (26.7)
	Stage III	41 (34.2)
	Stage IV	29 (24.2)
Histological grade	Well differentiated	44 (36.7)
	Moderately differentiated	54 (45.0)
	Poorly differentiated	22 (18.3)
Treatment modality	Surgery alone	26 (21.7)
	Surgery + radiotherapy	48 (40.0)
	Surgery + chemoradiotherapy	46 (38.3)

In total, 34 (28.33%) patients with recurrence exhibited significantly higher serum levels of EGFR, IL-6, and TNF-α at baseline, 6 months, and 12 months than those without recurrence (p < 0.001). This consistent elevation across time highlights their potential role in predicting disease recurrence (Table 2).

**Table 2:** Comparison of serum biomarker levels between patients with and without recurrence. Values are presented as mean ± standard deviation. *: P-values <0.05 are considered statistically significant. Comparisons between groups were performed using the independent samples t-test. Normality of data was assessed before analysis. TNM: tumor-node-metastasis staging system; EGFR: epidermal growth factor receptor; IL-6: interleukin-6; TNF-α: tumor necrosis factor-alpha; CI: confidence interval

Biomarker	Recurrence (n = 34)	No recurrence (n = 86)	Mean difference	95% CI	t-value	P-value
Baseline						
EGFR (ng/mL)	82.3 ± 24.1	62.9 ± 19.4	19.4	10.2–28.6	4.28	<0.001*
IL-6 (pg/mL)	26.4 ± 10.8	15.9 ± 7.1	10.5	6.7–14.3	5.62	<0.001*
TNF-α (pg/mL)	42.7 ± 16.3	28.6 ± 12.1	14.1	8.3–19.9	4.89	<0.001*
At 6 months						
EGFR (ng/mL)	54.8 ± 17.6	37.1 ± 11.2	17.7	11.8–23.6	5.96	<0.001*
IL-6 (pg/mL)	13.2 ± 6.4	6.9 ± 3.8	6.3	4.2–8.4	5.88	<0.001*
TNF-α (pg/mL)	26.1 ± 10.2	15.4 ± 6.1	10.7	7.1–14.3	5.92	<0.001*
At 12 months						
EGFR (ng/mL)	51.4 ± 16.1	33.5 ± 9.8	17.9	12.4–23.4	6.47	<0.001*
IL-6 (pg/mL)	11.8 ± 5.7	5.2 ± 3.1	6.6	4.6–8.6	6.54	<0.001*
TNF-α (pg/mL)	23.4 ± 9.1	12.0 ± 5.2	11.4	8.0–14.8	6.71	<0.001*

Correlation analysis demonstrated significant positive associations between the baseline biomarker levels and clinicopathological parameters. EGFR, IL-6, and TNF-α levels were strongly correlated with tumor size, TNM stage, histological grade, and lymph node metastasis (p < 0.001). Additionally, intermarker correlations were significant, particularly between IL-6 and TNF-α (r = 0.72), indicating a shared inflammatory pathway (Table 3).

**Table 3:** Correlation of baseline serum biomarkers with clinicopathological parameters. Correlation analysis was performed using Spearman’s rank correlation coefficient. *: P-values <0.05 are considered statistically significant. — indicates that diagonal elements representing self-correlations are not displayed. TNM: tumor-node-metastasis staging system; EGFR: epidermal growth factor receptor; IL-6: interleukin-6; TNF-α: tumor necrosis factor-alpha; CI: confidence interval

Parameter	EGFR		IL-6		TNF-α	
	r	P-value	r	P-value	r	P-value
Tumor size (cm)	0.54	<0.001*	0.48	<0.001*	0.42	<0.001*
TNM stage	0.61	<0.001*	0.57	<0.001*	0.51	<0.001*
Histological grade	0.38	<0.001*	0.44	<0.001*	0.36	<0.001*
Lymph node metastasis	0.52	<0.001*	0.49	<0.001*	0.45	<0.001*
IL-6	0.67	<0.001*	—	—	0.72	<0.001*
TNF-α	0.58	<0.001*	0.72	<0.001*	—	—

Table 4 presents the results of a multivariable Cox proportional hazards regression analysis for recurrence-free survival. The model was adjusted for age, sex, TNM stage, histological grade, and baseline and six-month biomarker levels (EGFR, IL-6, and TNF-α). For categorical variables, the following reference categories were used: male for sex, TNM stage I-II for tumor stage, and well/moderately differentiated for histological grade. Baseline EGFR (HR = 1.31, p < 0.001), IL-6 (HR = 1.28, p = 0.002), TNF-α (HR = 1.19, p = 0.028), and advanced tumor stage (HR = 2.18, p = 0.018) were significantly associated with increased recurrence risk. These findings highlight the prognostic value of circulating markers in OSCC (Table 4).

**Table 4:** Cox proportional hazards regression for recurrence-free survival. Multivariable Cox proportional hazards regression analysis adjusted for age, sex, TNM stage, histological grade, baseline EGFR, IL-6, TNF-α, and 6-month EGFR and IL-6 levels. Reference categories: male (sex), TNM stage I–II, and well/moderately differentiated histological grade. *: P-values <0.05 are considered statistically significant. TNM: tumor-node-metastasis staging system; EGFR: epidermal growth factor receptor; IL-6: interleukin-6; TNF-α: tumor necrosis factor-alpha; CI: confidence interval

Variable	Hazard ratio	95% CI	Wald χ²	P-value
Age (per year increase)	1.02	0.98–1.06	1.12	0.287
Sex (female vs. male)	0.84	0.41–1.72	0.24	0.624
TNM stage (III–IV vs. I–II)	2.18	1.14–4.17	5.62	0.018*
Histological grade (poorly vs. well/moderately)	1.89	0.96–3.72	3.41	0.065
Baseline EGFR (per 10 ng/mL increase)	1.31	1.12–1.53	12.84	<0.001*
Baseline IL-6 (per 5 pg/mL increase)	1.28	1.09–1.50	9.76	0.002*
Baseline TNF-α (per 10 pg/mL increase)	1.19	1.02–1.39	4.82	0.028*
6-month EGFR (per 10 ng/mL increase)	1.42	1.18–1.71	14.21	<0.001*
6-month IL-6 (per 5 pg/mL increase)	1.36	1.11–1.67	9.14	0.003*

## Discussion

The present prospective cohort study evaluated the prognostic significance of serial serum EGFR, IL-6, and TNF-α levels in OSCC patients. The findings demonstrated that elevated baseline and follow-up levels of these circulating factors were significantly associated with recurrence, advanced disease characteristics, and reduced recurrence-free survival. These results underscore the importance of integrating molecular and inflammatory markers into prognostic assessments beyond conventional clinicopathological parameters.

In this study, patients who developed recurrence exhibited consistently higher levels of EGFR, IL-6, and TNF-α at all time points. This observation supports the concept that persistent activation of growth factor signaling and systemic inflammation contribute to tumor progression and therapeutic resistance. EGFR is a well-established driver of tumor proliferation, angiogenesis, and metastasis, and its overexpression has been associated with aggressive tumor behavior and poor survival outcomes in head and neck cancers [9]. Previous studies have similarly demonstrated that elevated EGFR expression correlates with disease progression and resistance to therapy [16,17].

IL-6 and TNF-α are key pro-inflammatory cytokines involved in tumor microenvironment modulation. In the present study, both markers showed strong associations with tumor size, TNM stage, and lymph node metastasis. These findings are consistent with earlier reports indicating that IL-6 promotes tumor growth and immune evasion through the activation of oncogenic pathways such as JAK/STAT signaling, while TNF-α contributes to chronic inflammation, angiogenesis, and metastatic potential [8,10,18,19]. The strong correlation observed between IL-6 and TNF-α further highlights the presence of a shared inflammatory axis driving tumor aggressiveness.

An important strength of the present study was the longitudinal assessment of biomarkers. Unlike previous studies, which relied on single baseline measurements, this study demonstrated that elevated levels at 6 and 12 months were significantly associated with recurrence, suggesting that dynamic monitoring provides superior prognostic information. Persistent elevation of these markers after treatment may reflect residual tumor activity, micrometastatic disease, or an unfavorable tumor microenvironment, thereby explaining their strong predictive value [14].

Multivariate Cox regression analysis revealed that serum EGFR, IL-6, and TNF-α levels were independent predictors of recurrence-free survival, even after adjusting for conventional factors such as tumor stage and histological grade. This finding aligns with growing evidence that circulating molecular markers may outperform traditional staging systems in predicting clinical outcomes, as they better reflect underlying tumor biology [15,20]. The observation that tumor stage remained significant, while other variables showed weaker associations, reinforces the complementary role of biomarkers in refining prognostic models.

Clinical implications

The clinical implications of our findings are noteworthy. First, serum EGFR, IL-6, and TNF-α can serve as non-invasive prognostic tools for identifying high-risk patients at an early stage. Second, serial assessment may facilitate dynamic monitoring of disease status and could potentially aid in the earlier identification of patients at risk of recurrence during follow-up. Third, patients with persistently elevated levels may benefit from intensified surveillance, tailored therapeutic strategies, or inclusion in clinical trials targeting the molecular and inflammatory pathways. Incorporating these markers into routine clinical practice could enhance precision medicine approaches in oral oncology and improve patient outcomes.

Limitations

Despite its strengths, this study had several limitations. The study was conducted at a single center, which may limit the generalizability of the findings. Although the sample size was adequate for statistical analysis, larger multicenter studies are required for external validation. Additionally, while serial measurements were performed up to 12 months, a longer follow-up period would provide better insight into overall survival outcomes. The study also did not include receiver operating characteristic curve analysis to establish clinically applicable cut-off values. Furthermore, potential confounding factors, such as comorbidities and lifestyle variables, were not extensively adjusted for in the multivariate models. Although repeated-measures analysis or mixed-effects modeling could provide additional insights into within-subject temporal changes, the present analysis focused primarily on the prognostic relevance of biomarker levels at specific follow-up intervals. Future studies with larger cohorts, extended follow-up, and integrated molecular profiling are warranted to validate and expand upon these findings.

## Conclusions

The present study demonstrated that elevated serum EGFR, IL-6, and TNF-α levels were significantly associated with recurrence, advanced disease, and reduced survival in OSCC. Their persistent elevation during the follow-up highlights their value as dynamic prognostic indicators. These circulating factors independently predicted recurrence-free survival and provided additional insights beyond conventional clinicopathological parameters. Incorporating their assessments into routine clinical practice may facilitate early risk stratification, guide personalized treatment planning, and improve patient monitoring. Further large-scale, multicenter studies are recommended to validate these findings and establish standardized clinical thresholds.
